# *‘When I receive ARVs through my group*, *my heart settles’*: Participants’ perceptions and experiences of Friends for Life Circles for Option B+ in Kampala and Mityana Districts, Uganda

**DOI:** 10.1371/journal.pgph.0001326

**Published:** 2023-11-07

**Authors:** Joseph Rujumba, Rachel L. King, Joyce Namale-Matovu, Priscilla Wavamunno, Alexander Amone, Grace Gabagaya, Gordon Rukundo, Mary Glenn Fowler, Jaco Homsy, Janet Seeley, Philippa Musoke

**Affiliations:** 1 Department of Pediatrics and Child Health, College of Health Sciences, Makerere University, Kampala, Uganda; 2 Institute for Global Health Sciences, University of California San Francisco, San Francisco, CA, United States of America; 3 Makerere University-Johns Hopkins University (MU-JHU) Research Collaboration, Kampala, Uganda; 4 Department of Medicine, Johns Hopkins University, Baltimore, MD, United States of America; 5 London School of Hygiene and Tropical Medicine, London, United Kingdom; University of Toronto, CANADA

## Abstract

The Friends for Life Circles (FLC) was a parallel randomized controlled trial testing the efficacy of a group peer support intervention to support long-term adherence to Option B+ in Kampala and Mityana districts in Uganda. We explored FLC participants’ experiences and perceptions of the intervention on adherence to Option B+ for PMTCT and potential implications for strengthening the PMTCT program. We collected data from six focus group discussions with lactating women enrolled in the FLC intervention, and from 14 key informant interviews with health workers, district and national level stakeholders, as well as male partners of FLC participants. Data were analysed using a content thematic approach in a continuous and iterative process. Women described the FLC intervention as acceptable and beneficial in enhancing their understanding of HIV and the need for ART. The FLC helped women, especially those newly diagnosed with HIV infection to come to terms with their diagnosis and overcome the fear of death linked to testing HIV positive, and provided opportunities to enhance ART initiation, resumption and adherence. The FLC provided safe spaces for women, to learn about ART, and to receive support from peers including adherence reminders through home visits and ‘coded’ reminder messages. Receiving ART from support groups protected members from stigma and long lines at health facilities. Fear of stigma, health system challenges, the high cost of caring for animals and lack of money to save in groups were key challenges noted. The FLC support groups were crucial in providing needed support for women to initiate, resume and adhere to lifelong ART for Option B+. It is important that women who test HIV positive and start ART for life receive psychosocial support from peers and health workers to improve chances of preventing HIV transmission from mothers to children.

## Introduction

Mother-to-child transmission of HIV (MTCT) in sub-Saharan Africa is the leading cause of HIV infection in children. In 2012, the World Health Organization (WHO) updated the PMTCT guidelines and recommended that women identified with HIV infection during pregnancy and breastfeeding should immediately start antiretroviral therapy (ART) and continue for life (Option B+) [1]. Since 2012, Uganda adopted the Option B+ strategy and its rollout has continued to date [2].

Several programmatic benefits are associated with Option B+ [3]. These include high acceptability of the intervention and a reduced rate of MTCT with the rate of infants diagnosed with HIV in the first two months of life dropping to 1–3% [4, 5]. Viral suppression, increase in CD4 and reduction in mortality among mothers living with HIV are other key benefits of [6, 7].

Despite these benefits however, significant barriers limit Option B+ effectiveness in several settings especially in sub-Sahara Africa, a region with a high HIV burden [8]. These include: fear of HIV disclosure to sexual partners [9]; HIV related stigma at the individual, family, community and facility levels [10, 11]; discrimination, poor interaction with health workers [7]; fear of or experienced side-effects [12, 13]; as well as poverty and work related challenges [11]. Furthermore, significant loss to follow up of women initiated on Option B+ has been highlighted in some countries like Malawi, where 17% of all [14] Option B+ patients could not be traced six months after initiation of ART [15]. A qualitative study conducted in western Kenya, highlighted Option B+ specific challenges (same-day initiation into treatment, health care providers unconvinced of the benefits of Option B+, insufficient training); facility resource constraints (staff and drug shortages, long queues, space limitations); and lack of client-friendly services (scolding of patients, inconvenient operating hours, lack of integration of services, administrative requirements) as key barriers [16].

A cross sectional study conducted in Western Uganda in 2015 revealed that 36% of women initiated on Option B+ did not return to the health facility [17]. Health facility barriers have also been cited as contributing to poor adherence to Option B+, including inadequate access to early antenatal care, poor linkage between mother-baby pairs and postnatal health care services, lack of effective linkages between facility and community structures [18], poor systems to ensure long-term retention in care [19], and increased staff workload [7, 20]. A Tanzanian study exploring reasons for loss to follow-up among Option B+ patients highlighted health-related factors such as fear of or experienced medication side-effects, lack of HIV disease symptoms; psychological factors including loss of hope, HIV-related stigma; as well as socio-economic status such as financial constraints, lack of partner support, family conflicts, non-disclosure of HIV-positive status, and religious beliefs [21].

While studies in some resource-limited settings have shown better PMTCT intervention adherence outcomes with the use of community-based counseling, support groups and home visits [22, 23] as well as facility-based maternal support interventions, e.g. “mother2mother” mentoring programs [24, 25]. There is paucity of data on the effectiveness of support groups for PMTCT in Uganda.

Within the context of Option B+ program in Uganda, we designed and implemented a group peer support intervention to improve long-term adherence to Option B+ through a randomized parallel controlled trial titled, “Friends for Life Circles” (FLC), to improve retention in care and adherence to ART up to two years postpartum among women living with HIV receiving PMTCT Option B+ in Kampala (urban) and Mityana (rural) districts in Uganda [11, 26]. The FLC intervention participants were ART-naïve women living with HIV attending the PMTCT programme at Mulago and Mityana Hospitals and other health centers III and IV in Kampala and Mityana districts randomized to an enhanced group peer support intervention with income-generating activities (IGAs), while controls were pregnant women living with HIV attending the standard Ugandan Ministry of Health PMTCT programme at the same hospitals. We conducted an end line qualitative study and in this paper, we report FLC participants’ experiences and perceptions of the FLC intervention on adherence to Option B+ for PMTCT and we draw implications for strengthening the PMTCT program.

## Methods

### Study design and population

#### Parent study

The FLC for Option B+ was an open-label randomized controlled trial (RCT) in which we enrolled a total of 540 pregnant women living with HIV between May 2016 and May 2020 and randomized them 1:1 to an intervention group and a control group.

#### Intervention

The 270 pregnant women living with HIV enrolled in the intervention group were assembled into small groups of 8–10 members that were to meet on their own on a weekly basis in order to support each other physically (health wise), emotionally as well as economically through income-generating activities.

The primary outcome of the study was adherence to clinic appointments and to antiretroviral drugs. FLC group members were selected based on their ability to meet (area of residence) and their interest in a particular income-generating activity (IGA). Where participants residing in the same area were too few to form a group, they were assigned to groups as close as possible to their location. Participants were also given the opportunity to join other groups not necessarily in their area for reasons such as fear of stigma although these were very few (less than 1%). Besides the FLC participants, each group chose whether or not to invite their male partners to their group meetings However, few male partners (less than 1%) attended such meetings as most men linked these groups to pregnancy and child birth and considered them to be mainly for women. Meetings were held at a location of each group’s individual choice which included participants’ homes, churches, schools, community centers or the health facility. A study team comprising of a nurse counselor, an IGA consultant and IGA assistants, peers, and a community psychologist, conducted monthly meetings with each group at their preferred community hub. Peer mothers who were individuals with similar social background and/or life experiences as FLC participants provided support to women enrolled in the FLC intervention. Study counselors organized monthly FLC group meetings in which they provided ART and Cotrimoxazole to women, psychosocial support through individual and group counseling including on ART adherence while emphasizing peer support among participants. Participants who missed their follow-up visit were contacted by a study staff to reschedule their visit and those who were unreachable by phone were home visited by a study health visitor.

With support from the study team, FLC group members elected their leaders. During the monthly meetings, health education talks on topics such as drug adherence, nutrition, family planning, stigma and discrimination were conducted with emphasis on peer-to-peer support, sharing life experiences, and ART drug refills. In addition, each group chose an IGA for which they received training and start-up funds from the study. The IGAs initiated by FLC groups included goat rearing in Mityana district (rural), bakery (mainly involving making snacks), craft making (jewelry and sandals), liquid soap making, binding books and making charcoal briquettes for Kampala district (urban). Each group was trained in their local language (Luganda) in basic record and book keeping, leadership skills, financial management, marketing, quality management, customer care/relations among others, and later linked to the local community development office (under district local government) for group registration, additional financial and technical support for sustainability. In addition, the FLC groups were taken on exchange visits to other FLC groups as well as various institutions or NGOs with similar IGAs for learning purposes and motivation. Group members were encouraged to open up saving and lending schemes.

#### Standard of care

The participants in the Standard of Care (SOC) arm consisted of 270 pregnant women living with HIV attending the health facilities where they were enrolled from and received care routinely provided as per Uganda Ministry of Health guidelines for Option B+. SOC arm participants received counseling from clinic PMTCT counselors and collected their ART drug refills on an individual basis from the PMTCT clinics where they were enrolled. The SOC arm participants participated voluntarily in family support groups whenever available at their respective health facility as recommended by the Ministry of Health.

The Ugandan Ministry of Health guidelines for PMTCT were the main reference material in the provision of ART to study participants in both FLC and SOC.

#### Follow-up

Individual participants in both study arms were followed up for 24 months postpartum. FLC group activities were followed up until all group members reached 24 months’ post-partum.

### The qualitative end line study

The present qualitative study was conducted at the end of the study follow-up period in Kampala (urban) and Mityana (rural) Districts between August and October 2019. This study involved six focus group discussions and 14 key informant interviews that explored participants’ experiences and perceptions about FLC for Option B+ regarding clinic visit and drug adherence to the Option B+ strategy for PMTCT.

### Sampling and data collection

Data collection was phased, starting with FGDs followed by KIIs. This approach to data collection enabled researchers to probe for insights from one method of data collection to inform the subsequent data collection exercises. At the end of the six FGDs and 14 key informant interviews, saturation was reached, meaning no new insights were emerging and data collection was ended.

#### Focus group discussions (FGDs)

Overall, 45 women took part in the FGDs, 23 from Kampala and 22 from Mityana District as shown in Table 1. To select FLC groups to participate in FGDs, we considered variation in the performance according to the ranking criteria. The criteria included adherence to ART at 12 months post-partum visit, adherence to scheduled clinic appointment visits, average attendance of FLC group meetings, HIV disclosure, level of group friendship/cohesion (how well they knew each other, called or visited or supported one another, especially during crisis, how often they met on their own for group activities etc.), leadership ability and governance (how well the responsibilities of the group’s chairperson, treasurer, secretary and mobilizer were fulfilled) and success of the IGA (skills acquired such as marketing, becoming a trainer or trainees, record keeping and net income realized from the IGA).

**Table 1:** Characteristics of FGD participants, n = 45.

FGD Description	Number of participants
**Kampala site (Urban) n = 23**	
FLC group with best performance throughout the study	7
FLC group leaders from different groups	10
Women in standard of care	6
**Mityana site (Rural) n = 22**	
FLC group with the best practices	9
FLC groups that had not performed well throughout the study	7
FLC group that began with poor performance and later became successful	6

All FGDs were conducted in Luganda using a pre-designed FGD guide. Study participants from the selected FLC groups were called on phone or visited by the home health visitor, explained the purpose of this study and asked if they were willing to participate in the end of study FGDs. All accepted were invited for the discussion on a day and time convenient to the group members. The discussions in Kampala were conducted at Makerere University-Johns Hopkins University Research Collaboration (MUJHU Care) which is situated on Mulago Hospital complex in a quiet room reserved for this purpose. The FGDs in Mityana District, took place at Mityana Hospital in a quiet room reserved by the study team.

All discussions were conducted by two experienced qualitative researchers, one acting as a facilitator and the other as a note taker. These were not involved in delivering the intervention and provision of care to study participants. Topics for discussion included experiences forming and running group activities, factors that might have hindered or facilitated groups success, benefits of being part of groups, how FLC/peer support groups facilitated or hindered adherence to ART drugs for Option B +, and suggestions to strengthen the Option B+ program. On average FGDs 60–90 minutes.

#### Key informant interviews (KIIs)

Fourteen KIIs were conducted with health workers, policy makers, IGA trainers, male partners of FLC study participants and community leaders in order to document their perceptions and experiences regarding the FLC intervention. The details are shown in Table 2.

**Table 2:** Characteristics of key informants, n = 14.

Study Site	Category of participants	Number of participants
**Kampala**	Community development officer	1
	Community leader secretary for women	1
	Male partner for FLC participant	1
	Health worker medical officer	1
	Income Generation Activity Trainer	1
	MOH policy maker program officer PMTCT	1
	Male partner for FLC participant	1
**Mityana**	Community Development officer	1
	Income Generation Activity trainer	1
	Male partner of FLC participant	1
	Male partner of FLC participant	1
	MOH policy maker DHO	1
	Health worker Medical officer	1
	Community leader Secretary for women LC1	1

Key informants were selected purposively on the basis of their involvement in the planning and provision of PMTCT services or the FLC intervention. The first author called each of the identified KII, explained the purpose of the study and agreed on the time and venue for the interview which was either their office or Makerere University-Johns Hopkins University Research Collaboration (MUJHU Care) which is situated on Mulago Hospital complex for those in Kampala and Mityana Hospital for those in Mityana District. A pre-designed interview guide developed by the study team was used to conduct KIIs. Topics covered included experiences forming and running group activities for women, factors that might have hindered or facilitated groups success and suggestions to strengthen psycho-social support for women in the Option B+ program.

Interviews were done by JR in English or Luganda depending on informants’ preference and on average lasted 45–60 minutes.

In this paper we present findings from FGD participants’ and key informants’ on how the intervention enabled or hindered adherence to Option B+.

### Data management and analysis

To ensure rigor in data collection and data analysis, we made audio recordings of all of interviews and discussions. All audio files were transcribed and translated verbatim by a professional transcriber, who is a university graduate, does similar work for Makerere University-Johns Hopkins University Research Collaboration (MUJHU Care) on retainer basis and is proficient in both Luganda and English. Members of the study team checked all transcripts for completeness. Two co-investigators (JR and RK) with extensive experience in qualitative research methods read interview and discussion transcripts individually multiple times to understand the data and to identify emerging themes and sub-themes reflecting the experiences of study participants regarding the FLC for PMTCT intervention. The two researchers met several times to discuss the themes and sub-themes and to agree on a code book.

The transcripts were exported to Nvivo version 11 and coded by JR and RK using content thematic approach [16] in a continuous and iterative process. The two researchers had regular discussions of emerging issues. Results were synthesized based on study themes and discussed at a study team meeting. Direct quotations were selected and used in presentation of study findings. Findings from FGDs and those of key informants from Kampala (urban) and Mityana (rural) study sites were triangulated.

### Ethical approval and informed consent

The study was approved by the Joint Clinical Research Centre, Research and Ethics Committee, Uganda National Council for Science and Technology (UNCST–SS 3726), the Johns Hopkins University (JHM) IRB00076534 CR00031577 and the University of California San Francisco (UCSF) Ethics Committee IRB-176558. On the day of the FGD or interview, each potential study participant was taken through the objectives, methods, procedures, benefits and risks of participating in the study and assured that their decision to participate or not participate in the study would not affect the services they were to receive from the health workers or their employment. Those willing to participate in the study provided written informed consent before taking part in the FGD or interview.

## Results

**Fig 1:**
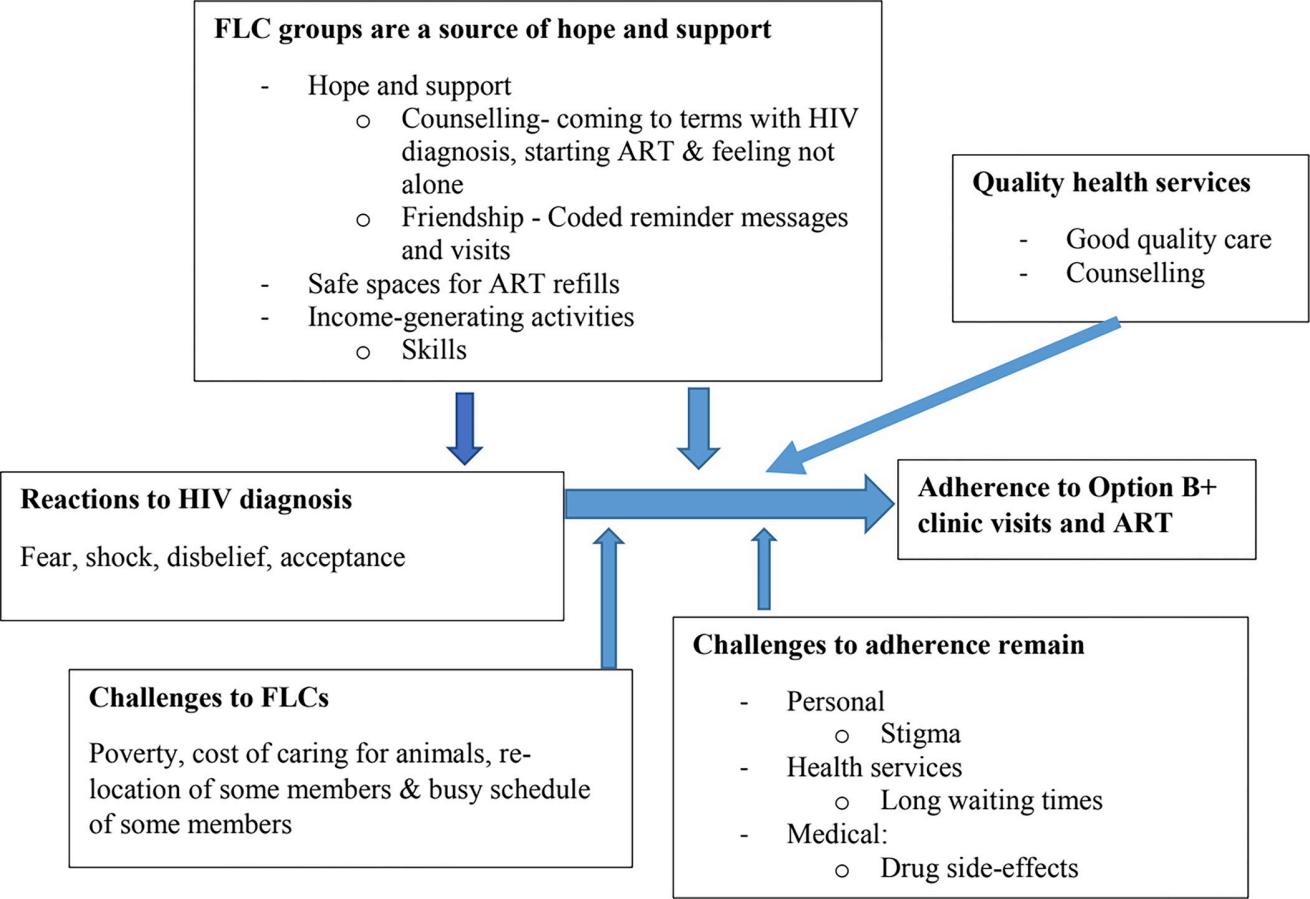
Thematic presentation of Friends for Life Circles (FLC) as enablers of adherence to Option B+ for PMTCT in Mityana and Kampala Districts.

Overall, FLC participants expressed positive experiences and general satisfaction with the intervention. Their experiences are summarized here under three themes: 1) FLC groups as a source of hope and support, 2) FLC members received quality health care and 3) challenges to adherence persisted. As shown in Fig 1, FLC intervention broadly facilitated women’s adherence to Option B+ by building on friendships initiated in their groups that enabled women to come to terms with their HIV diagnosis and to feel they were not alone living with HIV through shared experiences, visits and coded reminders among members that facilitated initiation and adherence to ARTs. As part of the FLC intervention, women reported receiving quality care from health workers that further aided adherence to Option B+. However, they reported that challenges of stigma, long waiting times and side-effects of ART continued to negatively affect adherence.

### FLC groups: A source of hope and support

#### Counselling—coming to terms with HIV diagnosis, starting ART and feeling not alone

The major theme from most narratives was that FLC groups were a source of hope and support for, especially those that were newly diagnosed, by enabling women to come to terms with HIV diagnosis and feeling they are not alone. Most women in the study mentioned that they were scared, shocked and in disbelief after receiving their HIV positive test result, some at their seventh and eighth pregnancy, and when they were asked by health workers to join the FLC peer support groups. Many women in both districts however mentioned that the counseling they received from health workers helped them to overcome the fear and they later joined the support groups. Some women noted that joining FLCs helped them to meet other women living with HIV, helping them to realize they were not alone. Also, women in FLC encouraged each other and gained courage to accept their new identity of living with HIV.

*We came here when some of us had fear*, *we feared to be with others …but now we are strong because we were counselled*, *joined colleagues*, *who would encourage us and together with health workers*, *they never let us down*, *they have been there for us up to now (FGD -FE02- Mityana*).

*You never saw me before*, *I hated myself*, *I never wanted to know anything*. *But this group has helped me*. *Also making friends and knowing that it is not only me who is like this [meaning being HIV positive] but there are many people*, *I got stronger (FGD- FE01-Kampala*).

Most women in both districts described their initial participation in FLC group meeting events as scary and worrying but later drew on encouragement from fellow women especially through realizing they shared an HIV-positive identity with others and thus they were not alone.

*In the first meeting*, *…I wasn’t myself because remember you have to be first tested and you get to know what is happening and you start the journey*. *But the health workers tried a lot to keep encouraging us*. *When I saw that I was with my fellow peers (women living with HIV) I said well*, *let me be strong at least it is not only me*. *But the first time it was not easy at all*, *I was fearing to die and I was so worried but I kept forgetting it because I was with my fellow peers whom I kept looking at and I would say “Even so and so is like this*, *let me get strong” (FGD-FE02-Mityana*).

Most women also mentioned that before joining the FLC groups they were worried about infecting their unborn babies, living life on medication and feared the ART drugs. But sharing experiences with other women living with HIV during FLC meetings especially those that had adhered to the eMTCT program and had given birth to HIV negative children helped them overcome these fears and carry on with taking ART.

*I used to fear a lot*, *I would be there and think that I am going to give birth to an HIV positive baby*. *But one of our members who gave birth first is the one who made me get strong when she said that she had given birth to an HIV negative baby*. *I also got relieved hoping that I will give birth to an HIV negative baby (FGD-FE02-Mityana*).

Indeed, some women explained that although they were given ART on the day they tested HIV positive as part of Uganda’s test and treat policy, they had kept or thrown away such drugs for fear of side-effects. Some study participants mentioned that they had been frightened by the large size of tablets and had delayed initiation or discontinued the drugs, but continued counseling from health workers, advice and encouragement from peers in FLC groups enabled them to start or continue their treatment. Many women drew on personal experiences taking ART including breaking tablets into two to make them easier to swallow, swallowing drugs after a meal and taking plenty of fluids to minimize side-effects of ART which enabled group members to start and adhere to HIV treatment.

*I first threw away the ART but later I said all my fellow peers are saying that let us take it (ART) to live longer and I started taking ART*. *I break the tablet into two parts and I take each at a go*. *The tablet later appeared small because I got used to taking it and it no longer causes any side-effects to me (FGD -FE02-Mityana*).

Some participants explained that FLC groups had helped them to overcome side-effects of ARVs in the initial period of starting ARVs but also to continue taking HIV medicines. Participants shared that they found the advice they received from group members such as taking medication after a meal, drinking adequate water and having sufficient rest was helpful in reducing the side-effects of ARVs especially in the early days of ART initiation. Most women revealed that whenever they thought about discontinuing ARVs owing to the side-effects, encouragement from group members and health workers helped them to continue.

*They tell us let us say you wake up in the morning and take your medication but it makes you feel bad inside your head and the health worker tells you that sometimes you have to drink a lot or get enough rest*. *Well*, *they advise you and you get courage within you and you take your medicine with courage (FGD-FE02-Mityana*).

#### FLC groups provided adherence support reminders- “coded” reminder messages, phone calls and visits by members

All women in the study reported that being part of FLC support groups was a source of encouragement and provided reminders which helped them to adhere to ART. Most women cherished the reminders they had received from group members about swallowing their drugs. Study participants revealed sharing reminders in the form of indirect or ‘coded’ messages that were only known by members of their specific FLC group. Some examples of indirect reminders mentioned include “have you taken water?” ‘remember water is life’, “have you taken juice?”, ‘have you loaded airtime’ among others. These “coded” messages worked to remind group members to adhere to treatment while maintaining confidentiality.

Study participants also mentioned receiving phone calls from FLC members or calling others to remind them about taking ART as some women explained.

*We are supposed to take it (ART) in time*, *you are not supposed to miss*. *And you have to call your colleagues and remind them*. *“Hey*, *it is time…” like me*, *I take my medicine at 08*:*00pm*. *I also have to be responsible to call Stephania (not real name) to ask how is it…*?*” We do not say it directly that medicine*, *we have a name we refer to it because she might be among other people*, *I can call and ask her “Are you done with taking your water*?*” and she will say “Yes*, *I am done” and maybe the other says “No it is not yet time…and the other calls the colleague too (FGD-FE01-Kampala*)

Use of social media was also mentioned as a medium used by FLC members to provide reminder messages to their colleagues.

*For me*, *Sarah has done a great job because whenever I check my WhatsApp*, *I find a voice message*. *Did you take your water*? *[Referring to ART]*. *I stay far*, *she cares a lot*. *Even others call “Did you take your water*? *Please ensure to take it*.*” (FGD-FE01-Kampala*).

Indeed, women and key informants acknowledged FLC groups as spaces that helped members share the challenges they faced as well as solutions related to adhering to ART.

*I got relatives (peers) that I never had*, *we are together*, *we talk well*, *we talk about all our challenges… (FGD-FE01-Kampala*).

Women mentioned visiting each other which gave them unique opportunities to share experiences, guide and learn from each other regarding coping with ART and addressing emerging needs related to living with HIV. Home visits by peers were also a source of hope. Through discussions and sharing, study participants reported that they would always find ways of addressing their problems.

*Home visiting encourages us so much*, *if you are seated somewhere and you have lost hope and your friends come to visit you*, *you talk about the situation you are going through*, *they encourage you even if you stopped attending group meetings—you still come back*. *Home visits are important and they empower us*. (FGD-FE02-Mityana).

*We started this group jokingly but the way we were mobilized*, *we didn’t know each other and some were coming from very far*, *but we started getting to know each other*, *we create each other’s happiness and we encourage ourselves on what we should do in our lives (FGD-FE01-Kampala*)

Another woman added how being a member of a support group had helped her access guidance from a colleague regarding care for her baby.

*We had met in one of our meetings and I got to know that she stays closer*. *So*, *when my child got sick*, *he had eaten soap*, *had a wound on his tongue and I got so scared*. *Remember we were told that you are not supposed to breastfeed when a child gets any wound in the mouth or on the tongue*. *So*, *when I showed her*, *because she had already completed breastfeeding*, *she knew more than me*, *she told me “go to the hospital*, *take him there”*. *I went and met the option B+ counsellor who guided me*… *(FGD-FE03-Kampala***).**

#### FLC groups as safe spaces for drug refills and time saving

Study participants reported positive experiences of receiving ART in their peer support groups compared to the general health facility. Women who had received ART from FLC support groups described these groups as safe places from stigma offering guaranteed privacy and confidentiality to members. Women noted that they saw and treated each other as friends, relatives and peers, thus reducing apprehension associated with receiving ART at general refill points in public hospitals where one could easily be identified as living with HIV by other people.

*When you go to the general ART unit*, *you find your friends and you do not want them to know your HIV status but here in the group*, *we were helped*, *you receive ART drugs from here and leave*, *no one gets to know* your HIV status (FGD-FE02-Mityana).

Another participant mentioned that receiving ART through FLC groups had saved them from gossip by other people at the health center. Receiving ART in FLC groups was also mentioned as a strategy to fight defeatism among study participants.

*Receiving drugs within the group has helped us to stop self-pity because some of us before we joined the group we were in a bad state*, *we would be there and feel self-pity …when we sit here together we stop having self-pity and get happy*… *(FGD-FE03-Mityana*).

Those who had returned to general ART drug refill points at health facilities recounted the challenges they experienced particularly related to fear of stigma.

*When we started receiving medicine through the group*, *I used to feel at peace*, *because I would arrive*, *talk to friends*, *get measured and receive drugs without going through any challenges*. *But at Kawempe [hospital] you get off the line and hide because someone at the maternity ward knows you and you don’t want her to see you*. *You hide and wait until she leaves for you to enter (FGD-FE01-Kampala*).

#### FLC aided skills building–income generation projects, leadership and vocational kills

Study participants reported that as part of being members of FLC support groups they had acquired new skills including starting and managing income generation activities, vocational skills such as baking, making exercise books, starting a restaurant and making shoes for those in urban areas or goat rearing for those in rural areas. Others reported learning leadership, communication, team work and interpersonal skills.

*They brought for us people from the sub-county and they trained us on how to manage a business*, *handling customers well*, *keeping time and marketing the business*. *…(FGD-FE01-Kampala*)

Some women mentioned that they had started income-generating activities as group members and others as individuals. While most of the business activities were newly established and had not started generating income at the time of this study, most study participants mentioned that these IGAs were a source of hope and were likely to yield income in the future and improve family welfare. Some women had started savings and credit associations in which group members saved and borrowed to meet individual and family needs including going for ART refills or start income generation activities.

Being part of the FLC groups was described as empowering to members. Some participants reported improved communication and interpersonal skills as well as confidence which enabled them to share their concerns openly, making it easy for the health care providers to attend to them.

*Support groups help women to open up and are able to discuss their challenges openly and get help…*. *When they come together like this*, *they share among themselves and even a service provider or a counsellor give them information*. *So*, *they are more likely to be more informed than a person who is not in the group on issues of health*, *nutrition*, *drug administration*, *HIV transmission and prevention* (District Official -KIE05-Mityana).

Some of the male partners mentioned the benefits to their wives being in FLC support groups. They reported that their wives acquired skills in managing businesses and were able to contribute to family requirements, without having to first request for money from their male counterparts. Some men reported improvements in family relationships given that they started sharing the responsibility for paying expenses.

*The other thing*, *it helps them develop themselves whereby you can earn some money and start a business which can support you so that you continue taking ART…* (Male Partner KIE03-Kampala).

Study participants were happy about the material support they received such as goats or items to start income-generating projects.

*I was given an animal*, *it gives birth to young ones*, *I am sure that when the group winds up*, *I will be having something that I can show people…*. (FGD-FE02-Mityana).

### FLC intervention members received dedicated and quality care from health workers

Study participants mentioned that they received dedicated care from health workers and the study team as part of the FLC study. This included being attended to swiftly, receiving counseling and follow-up on ART adherence, CD4 monitoring and guidance on how to care for their babies. Study participants noted that support from health workers and the study team through home visits, follow-up counselling and encouragement, provided understanding and care which were a source of encouragement for women to adhere to their treatment.

*I told health workers leave me*, *will I be the first to die*? *And they told me “no*, *take ART and you will see a difference” and right now I work well*, *I am happy*, *I do not have any problem*. *The other thing*, *the MUJHU people every after some time we come and they test us to see the CD4 count*. *It has also helped us because it makes us get strong that we have people who care about us* (FGD-FE02-Mityana).

Women in FLC intervention mentioned instances where health workers on the study team would go an extra mile to find drugs that were out of stock at health facilities, particularly ARVs and nevirapine syrup for babies.

Study participants also mentioned that healthcare providers conducted home visits and telephone calls to check on group members, especially those who missed group meetings or clinic visits. It was noted that women who needed drug refills but had missed clinic visits were visited at home by study team members who replenished their drugs.

*I have wiped out fear in myself*, *counsellors told me to take my medicine at the right time*. *I never wanted to take ART drugs*, *I had refused but one counselor called me on phone and asked “did you take your medicine*?*” I told her no and she said “take that medicine*, *be responsible*,.., *life is much better*…” *and that encouraged me (FGD-FE03-Kampala)*.

It was noted that some counselors especially peer counselors drew on their own experiences to provide support to other women especially those that had recently tested HIV positive.

*A health worker gave me an example that I should look up to her “I have also had such an experience…” and that strengthened me*. *If I hadn’t joined this study maybe right now*, *I wouldn’t be alive because at that time I looked for someone I could lean on and I did not have that person*, *but I remembered that I had counsellors in this study*, *I picked a phone*, *made a call and they invited me and counselled me*. *(FGD-FE03-Kampala*).

### Key challenges to adherence to Option B+ remain

Despite the numerous benefits reported by study participants regarding FLC support groups, several challenges persisted and threatened adherence to ART for option B+. These include fear of stigma associated with HIV and health facility challenges. Fear to be identified as living with HIV at health facility, community and family levels was the most common challenge mentioned by study participants as a threat to adherence to Option B+. At public health facilities, most women feared to be identified by their neighbors or relatives at ART drug refill points which would expose their HIV status.

Most women reported struggling to keep ART a secret in the context of ‘bottles’ that make noise in public transport, public places and at home. In all FGDs, study participants narrated how they struggled to silence the noise of ART bottles to avoid being labeled as being HIV positive. Study participants reported adding items like newspapers, cotton wool, pieces of cloth and toilet paper to silence the noise of ART bottles. Other participants removed drugs from bottles and packed them in other materials such as polythene bags and newspapers.

*When they give you ART in a tin it makes noise like shakers notifying others of who you are and that is why I leave the bottle home*. *We were told here that when you are coming for a refill you come back with the tin but we do not bring it because It makes noise…* (FGD -FE02-Mityana).

Non-disclosure of HIV status to male partners and other people in one’s social circles was another barrier to adherence to option B+. Not disclosing HIV status to family members makes adhering to medication more difficult as some participants described how they had to leave medicine behind when visiting family members. Women avoided disclosing their HIV status to male partner’s for fear of being blamed for bringing HIV in the family. Alcohol use and inability to afford food and water were discussed as a key issues in failure to adhere to ART. Side effects, especially dizziness, were also mentioned as deterrents of ART adherence.

#### Health system challenges

Some of the health system challenges mentioned included stock out of ART or of drugs that are intended to treat opportunistic infections, limited number and unfriendly health workers, and long queues resulting in delays at health facilities. Indeed, many women expressed fears about receiving ART from the usual health facility when the study comes to an end as these places are characterized by high number of patients, few health workers and lengthy waiting time.

### Intervention level challenges

At intervention level, poverty in form of food insecurity and lack of money to save in groups, the high cost of caring for animals, some group members relocating to distant places as well as the busy schedules among some members due to employment and domestic care demands were key challenges.

In all FGDs participants mentioned poverty characterized by food insecurity and lack of money to save in groups. As such, some group members infrequently participated in group meetings and other activities which in turn negatively affects group cohesion.

*When you do not have what to eat*, *you cannot get what to save in the group*. *This lack of money to save discourages some members from attending group meetings (FGD-FE01-Kampala*).

Another common challenge mentioned by participants in Mityana District was the high cost of caring for goats and lack of agricultural extension services especially when they fall sick which encroached on their meager resources.

*The challenge of animal diseases has been common and yet treatment is very costly*. *You treat the goat today and before the month ends it is sick again*. *Sometimes you have to borrow money to treat the goat and some of our members lost the goats …(FGD-FE02-Mityana*).

Some group members relocating to distant places because of finding a new job or family relocation was another challenge which increased the cost of transport to participate in group activities and made support in form of home visits by other FLC support group members difficult. Busy schedules at work or domestic responsibilities including care for children and husbands were other challenges to the success of group activities.

*Some of us we are employed*, *you cannot tell your boss that I am going for a meeting and then again*, *another meeting*, *in the same month*, *he can lose trust in you and eventually dismisses you (FGD-FE02-Mityana*).

*I am the chairperson of the group but I also have a job*, *I am employed in a company*. *… I cannot leave work for group activities all the time*. *Sometimes I arrive for group meetings late …(FGD-FE01-Kampala*).

*I have to first look after my husband*, *get what he will eat*. *I have to leave after getting what the children will eat and that delays me for group meetings (FGD -FE03-Kampala*).

## Discussion

In this study, the FLC intervention was positively appraised by all study participants for having helped them come to terms with their HIV diagnosis, changing their perception of HIV as a death sentence to an infection that one can live with, and providing opportunities to enhance ART initiation, resumption and adherence. Most FLC intervention participants felt that the support groups provided safe spaces for women, especially when newly diagnosed, to meet other women living with HIV which enhanced acceptance of self and generated courage to live on with HIV by making them aware they were not alone. Women who had lost hope and feared giving birth to HIV-positive babies were encouraged by the testimonies shared in their groups by other women living with HIV who had given birth to HIV-negative babies. FLCs created opportunities that enhanced ART initiation, resumption and adherence. Through sharing experiences, women who had feared ART were able to start and those who had discontinued ART were able to resume treatment. Group members provided adherence reminders through home visits and sending ‘coded’ reminder messages that were only understood by members of a specific group. Study participants that had received drug refills from their groups credited the approach for enhancing confidentiality, saving them from stigma and time-consuming line-up at congested health facilities. Participants also mentioned that the FLCs helped them access dedicated care from study staff and health workers as well as skills development in relation to leadership and income generation.

The experiences of women participants in this study show that despite over two decades of PMTCT and ART provision in Uganda, women who test HIV positive during pregnancy and breastfeeding period still face the fear of stigma, death and likely transmission of HIV to their children, and thus require enhanced counseling and support from peers and health workers to overcome and address these fears. As our findings have indicated, without immediate and continuous psychosocial support, some women do not initiate ART even when the drugs are provided at diagnosis, a practice that is likely to continue compromising the outcomes of Option B+. It is thus critical that PMTCT programs offer targeted individual and group support to women diagnosed with HIV to help them come to terms with their new diagnosis, regain hope to live and increase opportunities for adherence support.

Our study revealed that women sharing experiences through FLC groups helped members overcome the fear of death often associated with HIV. The implication here is that support groups provide valuable space for women to overcome feelings of being alone and enhances togetherness that was critical to overcome HIV stigma and other challenges encountered in adhering to ART for life. Although all women had been given ART, some had not started taking their drugs while others had discontinued the drugs owing to felt or feared side-effects. A recent study in Uganda, revealed that early adherence to Option B+ was sub-optimal due to non-disclosure, fear of side effects of ART and distant health facilities [27]. Another Ugandan study documented a higher risk of loss to follow-up among mothers in Option B+ who started ART on the day they tested HIV positive [28], reflecting a need for support. Joining the FLCs had helped women gain confidence to resume or start taking such drugs. Women members of FLCs drew on their own experience of taking ART to guide and re-assure others on the value of ART but also on the short-lived nature of side-effects and how to minimize them. Such experience sharing complemented and validated information women had received from health workers. These results show that it is important that program implementers pay attention to non-disclosure of HIV positive status, stigma and fear of side-effects of ART as prominent barriers in the early stages of HIV diagnosis and ART initiation in order to ensure that women are ready for lifelong ART adherence required under Option B+. The role of peers in promoting ART adherence is crucial especially for women who are newly diagnosed with HIV. A recent systematic review concluded that peer support groups for people living with HIV have positive effects on retention in care, ART adherence and viral load suppression [29]. An earlier study on roles of community cadres to support retention in PMTCT in four African countries including Uganda revealed that support networks provided mothers with emotional support, motivation and a platform to share knowledge and experiences [30].

Unique to our study is the use of ‘coded’ adherence reminders among FLC group members building on the friendship ties established in specific support groups. These ‘coded’ reminders were not planned as part of the study but instead grew organically, were unique to group members and were informal in nature, which made them more effective to support adherence but also cushioned members against HIV stigma. Such reminders could potentially be considered for text messaging and other mobile health interventions. Group members also observed that visits by members enhanced shared learning and mutual support.

Groups were also credited for being safe spaces from long lines and stigmatization during ART drug refills which are common barriers at public health facilities in Uganda. This finding demonstrates the potential and a need to scale-up Differentiated Service Delivery (DSD) models for HIV care in Uganda to save time and reduce congestion at health facilities. DSD has been defined as ‘a client-centered approach that simplifies and adapts HIV services across the cascade, in ways that both serve the needs of people living with HIV better and reduce unnecessary burdens on the health system [31, 32]. The DSD models have been documented to improve adherence, peer support, reduce stigma and patient travel costs in South Africa, Uganda and Zimbabwe [33].

Study participants also appreciated the dedicated care they received from the study team and were worried about going back to the main drug refill points at the hospitals characterized by congestion and long lines. This finding reflects the need for continued client feedback and the need to address the perpetual constraints faced at ART clinics including staff shortage and overload. These constraints have been widely documented in Uganda and affect program success [11, 34].

Skills building and income-generation projects were other benefits mentioned by study participants derived from FLC intervention. While most IGAs had recently been started (most were less than one year) and had not yet yielded income to members at the time of this study, participants had hope that these would generate income in future. Some groups had started village savings and loan associations and had borrowed from such groups to meet family needs. Interventions addressing economic insecurity have potential to bolster health outcomes by improving ART adherence in low resource settings [35].

Despite the numerous benefits reported by study participants regarding FLC support groups, fear of stigma associated with HIV, stock out of ART, lack of food, few and unfriendly health workers, congestion and delays at public health facilities persisted as challenges and threatened to hinder adherence to ART for option B+ [11]. In part these challenges depict the need for health system strengthening to better deliver the Option B+ package as has been documented in other studies [8, 34, 36]. Similar to our findings, a recent meta-ethnography of qualitative research from sub-Saharan Africa, highlighted adequate interactions with health workers, persistent stigma exacerbated by space constraints in health facilities and drug stock-outs as consistent health system constraints for women’s engagement with Option B+ services [8]. Taken together, these findings imply that national and international stakeholders aiming at supporting the elimination of MTCT of HIV need to pay more attention to addressing these health system challenges. Poverty in form of food insecurity and lack of money to save in groups, the high cost of caring for animals in the context of limited agricultural extension services, some group members relocating to distant places as well as the busy schedules among some members due to employment and domestic care demands were key challenges to successful implementation of the FLC intervention. These should be of interest to stakeholders seeking to implement similar interventions.

Our study findings should be understood in light of the following strengths and limitations. Use of qualitative methods of data collection facilitated an in-depth understanding of women in FLC peer support groups and stakeholders involved in the planning and provision of PMTCT services. Use of FGDs and key informant interviews in the study enabled triangulation of data thus improving the trustworthiness of our findings. However, data were collected within the premises of a randomized controlled trial which has potential for bias in the experiences reported by our participants. The data collection team which was independent from the study implementers assured participants of confidentiality and the need for them to be open about their experiences of being part of the FLC intervention. Besides, in all discussions study participants openly expressed their opinions often comparing and contrasting the care received at public health facilities and in the study. In addition, the narratives of women in the two districts and those of key informants regarding FLC intervention reflect agreement which is reassuring about the validity of our findings. The type of FGDs varied by levels of group performance in the two study sites which limited comparisons across groups. Besides, few men participated in FLC and in this study. Thus Male partner reports regarding FLC intervention for Option B+ are based on a few men owing to their limited participation in the parent study. Thus future studies should deliberately strive to increase male partner participation to better understand how women’s income generation affects relationships with male partners. At the time of the study, most income-generating activities had recently been initiated and thus it was not possible to assess their contribution towards alleviating the socio-economic barriers often faced by women in adhering to Option B+ and general HIV care. Future studies that allow adequate time for income-generating activities to mature and assess their impact on adherence to HIV care are recommended. In addition, women in our study expressed apprehension about being referred back to the general HIV clinics at the end of the study, reflecting a need for studies to understand post-intervention dynamics and experiences of study participants referred back to general health care settings especially at public health care facilities with human resource and medical supply constraints. Follow-up studies would also aid exploration about sustainability barriers and enablers of such interventions.

## Conclusion

Pregnant and lactating women enrolled in the Friends for Life Circles intervention perceived the intervention as acceptable and beneficial in enhancing their understanding of HIV and the need for ART. The intervention helped women especially those newly diagnosed as HIV positive to come to terms with their HIV diagnosis, overcome the fear of death linked to testing HIV positive and facilitated initiation or resumption and adherence to ART. It is important that pregnant women who test HIV positive receive psychosocial support from peers and health workers to improve chances of preventing HIV transmission from mothers to children and realize other benefits of ART for life. Addressing health system and structural challenges including stock-out of critical supplies for HIV care, staff shortage, space constraints and stigma is critical for success of such interventions aimed at eliminating MTCT of HIV. The success of agricultural related income generation activities such as goat rearing requires strengthening agricultural extension services by government and development partners. Linkage of such groups to other poverty alleviation and household economic strengthening interventions is critical for participants to meet household needs and increase earnings to effectively participate in group activities including savings. In addition, support groups will need to adjust their schedules to avoid conflict with work and domestic roles of members.
